# The *Verulam Review* and the British Medical Association

**Published:** 1900-05-12

**Authors:** 


### THE *VERULAM REVIEW* AND THE BRITISH MEDICAL ASSOCIATION.

The British Medical Association is to be congratulated on the result of the action for libel brought against it by the editor of the *Verulam Review*. Although we entirely fail to see how any one who had written such letters as were shown in the trial to have been written by the plaintiff could ever have persuaded himself that he had any proper cause of complaint against the *British Medical Journal*, for the very moderate comment on his proceedings which had appeared in that journal, juries are proverbially skittish in libel cases, and there always was the chance that some prejudice might be imported into the case by its connection with the vivisection question in regard to which the dispute arose. It is very satisfactory, then, to find that the verdict was for the defendant, with costs. It is not, however, in its relation either to the plaintiff or the defendant that the interest of this trial centres so far as the outside world is concerned, but rather in the fact that opportunity was given in its course for witnesses to state on oath certain facts in regard to which much ignorance and misconception exist among some, and as to which erroneous assertions are often made by others. Dr. Vivian Poore repeated on oath the statement made in his Report for 1897, that "the only experiments performed without anæsthetics are of the nature of inoculations or hypodermic injections." A great deal of the evidence centred round the so-called "Certificate A," under which operations can be performed without anæsthetics, and it was brought out in evidence with the greatest clearness that this certificate must bear the nature of the operation for which it is granted on the face of it, and that these certificates never have been used for "serious operations other than inoculation." Professor Sims Woodhead, who has been for a long time at the head of a large laboratory, said that "leaving out inoculation experiments he had not seen in his laboratory an experiment upon a living cat or dog without an anæsthetic"; and, further, that "he had no experience of any painful experiments upon animals in this country without anæsthetics." People are so constantly being told of the "horrors" that are going on in regard to experiments upon animals that they find it difficult to believe that most of what they hear is all moonshine. Perhaps, now that the above statements have been made on oath in a Court of Justice, they will be more satisfied.

